# A Treatise on the Nature and Virtues of the Saline Spring at Moira Baths, near Ashby-de-la-Zouch, Leicestershire; to Which Are Added, Remarks on the Use of Warm and Cold Bathing

**Published:** 1817-08

**Authors:** 


*A Treatise on the Nature and Virtues of the Saline Spring at Moira Baths, near Ashby-de-la-Zouch, Leicestershire; to which are added, Remarks on the Use of warm and cold Bathing.* By JAMES ARDEN, M. D. formerly of Trinity College, Oxford. 12mo. pp. 91. Burton-upon-Trent, 1817.

ALTHOUGH utterly strangers to Dr. Arden, we had imbibed, from the report of a writer connected with this Journal, no ordinary, and we are *yet* inclined to hope, no incorrect opinion of his character as a man of letters, a philosopher, and physician. Consequently, amid the present dearth of interesting productions in the field of literature, we unbound the packet, enclosing his treatise from the "Row," with feelings of eager expectation, which served but to aggravate the subsequent disappointment: for that we are woefully disappointed in the perusal of Dr. Arden's work, truth constrains us to acknowledge.

Most of our readers are probably aware, that, some years since, a spring of water, strongly impregnated with saline substances, was discovered, in the operation of digging for coal, near Ashby-de-la-Zouch, in Leicestershire. An account of its chemical analysis by Mr. Accum was recently published in our Journal.* To explain the properties and illustrate the employment of this water in the cure of diseases, constitutes the professed object of Dr. Arden in writing the present Treatise.

But as Dr. Arden confessedly adopts the analytical report of Mr. Accum with respect to the composition of the Moira water; as there is nothing advanced upon its medicinal uses, which every mature practitioner, and indeed

---

* See *Medico-Chirurgical Journal,* vol. ii. page 352.

every young medical gentleman who has happily gone through the process of molar dentition, may not directly infer from a retrospect of its chemical history; and as the work, from its style, is adapted rather for the general than professional reader, we shall decline entering into any regular analysis of its contents, and thus spare ourselves the trouble, and Dr. Arden the pain, of which such a scrutiny must have been productive. To a few cursory remarks on the medical doctrines, physiology, and chemistry, of the Treatise, and on the importance of a correct style of composition in medical writings, we shall therefore restrict ourselves. Dr. Arden will, we hope, listen to our admonitions in a spirit as liberal as that by which they are dictated, and turn them to account in his future writings. That he is yet young, we have every reason to believe: we know that he possesses talents of no ordinary description; and we intreat him ever to bear in mind the responsibility which devolves upon those to whom such talent is entrusted. And may he, the Byron of medical literature, expiate the sins of his youthful enterprize, and obliterate the recollection of his failure, by the number and unrivalled excellence of the productions of his maturer age!

The *Practical Observations* of Dr. Arden constitute by far the most valuable portion of his Treatise; they are generally correct, and evince sound observation, and a respectable acquaintance with the productions of the earlier writers on medicine.

His *Physiology* is rather of an antiquated cut. The absorption of the oxygen of the atmosphere by the pulmonary blood, the production of animal heat by its subsequent extrication, and the dependence of the red colour of the vital fluid upon its combination with iron,* are heresies which have long been erased from the page of physiological science by the hand of British genius, and which we little expected to have seen revived in the work of such a man as Dr. Arden.

Yet worse is the *chemical portion.* That *Epsom salt* might have been misnamed *muriate* of magnesia through the negligence of blundering printers, men frequently more stupid than the very devils whom they employ; and that such error might have eluded the vigilant eye of Dr.

* See an important paper on the *colouring Principle of the Blood of Animals.* Medico-Chirurgical Journal, vol. ii. p. 508.

Arden, amid the pressure of his other engagements, we could readily imagine, and were willing to believe. But, how the term *azote* became prefixed to a description of the chemical characters of *carbonic acid gas*, we profess ourselves utterly unable to comprehend. We have in vain looked through *Parke's catechism*, from which most of the chemical information, and much of the poetical embellishment of the treatise, have evidently been borrowed, in the hope of tracing to its source this most curious and unfortunate blunder. Over the less conspicuous and important blemishes, we pass in silence, merely observing that, as the experiments of Dr. Arden on the Moira water, with different re-agents, are neither sufficiently numerous nor precise, to confirm or refute the accuracy of Mr. Accum's analysis, we see not the policy of swelling the Treatise by their recital.

Lastly, with respect to *composition*, the book is exceedingly defective. Were it necessary to demonstrate the accuracy of this opinion, there is scarcely a page which would not furnish several examples in confirmation of it. Yet, although determined, for our own sake, as well as that of Dr. Arden, not to let such negligence pass wholly without correction, we are unwilling to dilate on what may be deemed an invidious topic. By some, indeed, it has been asserted, that correctness of style in medical writings is a matter of little consequence; nor is such opinion, abstractedly viewed, destitute of a certain degree of plausibility: for no one can doubt that the unlettered practitioner will frequently evince the same tact in the diagnosis of a disease, and quite as much ability in its treatment, as the most profound and accomplished scholar. A similar remark is equally applicable to the honours of medical graduation. Neither the general erudition, nor the degree, is *essential* to the constitution of the medical character. But are such attainments, therefore, to be spurned and rejected? Unquestionably not. To the possessor they are at least valuable, as certificates that he has regularly gone through that course of general and professional education by which alone he can be fitted for the elevated functions of his calling, and for the honourable rank and character which the physician ought to hold in the scale of society; in fact, as the exterior and palpable signs by which the unprofessional world around him may be enabled to form a general opinion of his deeper and more difficultly appreciable qualifications and acquirements. And few reflecting persons would, we presume, estimate very favourably the abilities of a physician who wrote not the language of his own country with tolerable purity and correctness. Thus viewed, then, the negligence of Dr. Arden, in the structure of his Treatise, is an act of injustice to himself, as glaring as it is wilful, and inexplicable upon any principle of human conduct or policy with which we are acquainted. That the defect is *wholly attributable to negligence,* appears most certain; for, although not fortunate enough to have heard or read any of his efforts or productions in this way, we learn that Dr Arden is an able orator, and has written on other subjects with a spirit and eloquence highly honourable to his character as a man, and to his attainments as a scholar.

